# Prevalence and Risk Factors Associated with Low Back Pain in the Saudi Adult Community: A Cross-Sectional Study

**DOI:** 10.3390/ijerph182413288

**Published:** 2021-12-16

**Authors:** Ahmed S. Alhowimel, Faris Alodaibi, Mohammed M. Alshehri, Bader A. Alqahtani, Mazyad Alotaibi, Aqeel M. Alenazi

**Affiliations:** 1 Department of Health and Rehabilitation Sciences, Prince Sattam Bin Abdulaziz University, Alkharj 11942, Saudi Arabia; dralqahtaniba@gmail.com (B.A.A.); maz.alotaibi@psau.edu.sa (M.A.); aqeelalenazi.pt@gmail.com (A.M.A.); 2 Department of Rehabilitation Science, King Saud University, Riyadh 11362, Saudi Arabia; falodaibi@ksu.edu.sa; 3 Physical Therapy Department, Jazan University, Jizan 45142, Saudi Arabia; phdalshehri@gmail.com

**Keywords:** low back pain, prevalence, community, risk factors

## Abstract

Worldwide, low back pain is common and linked with many risk factors. There is a lack of studies in the Saudi Arabian community on low back pain prevalence and risk factors. Therefore, the present research will investigate the prevalence of low back pain in the middle-aged and elderly community population and examine the risk factors contributing to low back pain in Saudi Arabia. The present paper is a cross-sectional study of the community living in Riyadh and the surrounding areas in Saudi Arabia. Data were collected between October 2019 and April 2020 via trained research assistants. A total of 276 participants were included in the analysis. The prevalence of low back pain was 27.9% (*n* = 77) among the participants included in this study. All participants reported low back pain severity with a mean of 4.35 ± 2.19 on the pain rating scale. Older age, arthritis, hypertension, anemia, osteoporosis, and a history of fractures were all associated with having LBP. Low back pain is highly prevalent in the Saudi community adult population living in Riyadh and its surrounding areas. More than a fourth of the sample reported experiencing back pain. The study outlines several modifiable risk factors (BMI, education, employment status, marital status, and smoking status) and unmodifiable risk factors (arthritis, hypertension, anemia, osteoporosis, and a history of fractures) associated with low back pain in the study sample.

## 1. Introduction

Around 50–80% of individuals suffer from Low back pain (LBP) at some point in their lives [1,2]. It is estimated that people over the age of 18 who can work are most susceptible to LBP, which is, globally, the most critical reason for disability [3,4]. The burden of LBP stemming from work-related ergonomic exposures was projected to result in about 21.8 million disability-adjusted life years (DALYs), in 2010 (95% confidence interval (CI) 14.5–30.5) [3]. Nearly everyone will suffer from lower back discomfort at some point in their lives [2]. The prevalence of the 1 year period generally occurs in adults worldwide and increases when one reaches the middle-age period. Women are more likely to have LBP than men. People with low back discomfort who cannot engage in everyday activities have a greater risk of developing such conditions with age [5].

Despite the uncertainty in the literature, research shows that the frequency and incidence of LBP in the Saudi Arabian general population, and the risk factors linked with LBP, are not well-defined and vary among studies. Therefore, most of the findings are drawn from research analyzing work-related musculoskeletal conditions [6].

Numerous individual and environmental risk factors for LBP have been identified [7,8]. For example, individual factors, such as metabolism, biochemistry, physical factors, and depressive tendencies, have been identified as risk factors for LBP [6]. Moreover, environmental risk factors, such as job satisfaction, lifting heavy weights, prolonged standing, forward bending, and carrying school backpacks, have been identified [7]. However, there is limited evidence of other associated risk factors of LBP, such as arthritis, diabetes, hypertension, cardiovascular diseases, dyslipidemia, anemia, osteoporosis, and the history of fractures, in the Saudi population.

There is a paucity of research in the Saudi Arabian population about the prevalence and risk factors of LBP. To our knowledge, there has never been a community-based cross-sectional study assessing the prevalence of LBP in Saudi Arabia. Therefore, the present study will explore the prevalence of low back pain among middle- and elderly-aged Saudi community living in Riyadh and its surrounding areas, and will examine the risk factors associated with LBP.

## 2. Methods

### 2.1. Design and Participants

The present work was a cross-sectional study of the community living in Riyadh and the surrounding areas in Saudi Arabia. Data were collected between October 2019 and April 2020 via six trained research assistants who were pre-trained by the research team. The data was acquired through an in-person survey. The study team examined each survey for any missing data. Participants who were aged 40 years and older, able to read and write in Arabic independently, and follow verbal commands, were included in this study. Recruitment was conducted through advertising in the media and in the local community, as well as through the collaboration with local residential communities (i.e., social centers and residential district committees). The present study was approved by the Research Ethics Committee (No. RHPT/019/031) from Prince Sattam Bin Abdulaziz University, Al-Kharj, Saudi Arabia. All participants who agreed to participate signed an informed consent before the start of the data collection.

### 2.2. Demographics

A designed form for data collection was reviewed and approved by the research team before the data collection. A survey form was utilized to obtain data on age in years, sex (male or female), weight, height, marital status, education, occupation, and smoking status. Weight in kilograms and height in meters were recorded via self-report to calculate the body mass index (BMI) by dividing mass in kg by height in m. Four categories for determining marital status were used, including single, married, divorced, and widowed. The educational level was obtained via self-report and categorized into none, elementary school, middle school, high school, and university level. Three categories for occupation were used, including unemployed, employed, and retired.

### 2.3. Chronic Conditions and Measures

Comorbidities were obtained via self-report using a structured interview. Each participant was asked about chronic conditions, and the answer was recorded as “yes” or “no”. Chronic conditions included arthritis, diabetes, hypertension, cardiovascular diseases, dyslipidemia, anemia, osteoporosis, and a history of fractures.

### 2.4. Outcome Measures

The main outcome measures for this study were back pain measured by the visual analog scale, by which participants rated their pain between “no pain” (score of 0) and “worst imagined pain” (score of 10). The definition of back pain is pain that is felt below the costal margin and above the inferior gluteal folds, either with or without radiating lower limb symptoms.

### 2.5. Statistical Analysis

The primary outcomes for this study were the presence of low back pain and low back pain severity using a numeric rating scale. Comparisons between people with or without low back pain were made using chi-square for categorical variables and the Mann–Whitney test for non-normally distributed data. To determine the association between risk factors and the presence of low back pain (yes vs. no), multiple binary logistic regression analysis was used. Odds ratio (OR) along with 95% confidence interval (95% CI) were calculated. The final model was reported here, including the factors entered as predictors (i.e., arthritis, diabetes, cardiovascular diseases, hypertension, dyslipidemia, osteoporosis, and a history of fractures) and the presence of low back pain (yes or no) as the dependent variable. All analyses were adjusted for covariates, including age, sex, BMI, marital status, education, employment status, and smoking. The primary analysis adjusted for covariates included age, sex, BMI, marital status, education, employment status, and smoking. An alpha level of 0.05 was used for all analyses. All analyses were performed using IBM SPSS for Mac version 25.0 (SPSS Inc. Chicago, IL, USA). 

## 3. Results

A total of 276 participants were included in the analysis. The prevalence of low back pain was 27.9% (*n* = 77) among participants included in this study. Low back pain severity was reported by all participants with a mean (4.35 ± 2.19). The demographic characteristics and clinical variables for participants with and without low back pain are shown in Table 1. Of these risk factors, only aging, the presence of arthritis, hypertension, anemia, osteoporosis, and a history of fractures were significantly different between people with and without a low back.

The results of the binary logistic regression examining the association between risk factors and low back pain are shown in Table 2, along with the odds ratio (OR) and associated 95% confidence interval (95% CI). Individuals with low back pain were more likely to have arthritis (OR 2.47, 95% CI (1.12, 5.32), *p* = 0.048); hypertension (OR, 2.34 95% CI (1.08, 5.05), *p* = 0.030); anemia (OR, 7.27, 95% CI (2.06, 25.69), *p* = 0.002); osteoporosis (OR, 5.94, 95% CI (1.63, 21.69), *p* = 0.007); and a history of fractures (OR 3.93, 95% CI (1.83, 8.44), *p* < 0.001), when compared to those without low back pain.

The results of the multiple linear regression analysis to examine the association between risk factors and low back pain severity are shown in Table 3.

The results showed that arthritis was significantly associated with increased lower back pain severity (B = 1.31, 95% CI (0.51, 2.12), *p* = 0.001) after controlling the other covariates, including age, sex, and BMI status, education, employment status, and smoking. Another factor (hypertension) was associated with increased low back pain severity (B = 1.11, 95% CI (0.28, 1.95), *p* = 0.009) after controlling the other covariates. Anemia was associated with increased low back pain severity (B = 2.11, 95% CI (0.85, 3.36), *p* = 0.001) after controlling the other covariates. Osteoporosis and a history of fractures were associated with increased low back pain severity (B = 2.50, 95% CI (1.18, 3.81), *p* = 0.001, and B = 1.67 (0.87, 2.48), 95% CI (0.87, 2.48), *p* = 0.001, respectively), after controlling the other covariates.

## 4. Discussion

This study aimed to explore the prevalence of LBP among the middle-aged and elderly community and examined the risk factors associated with LBP in Riyadh, Saudi Arabia. The prevalence in these community-based middle-aged adults was about 28%, and there were several associated comorbidities with the existence of LBP. Older age, arthritis, hypertension, anemia, osteoporosis, and a history of fractures were all associated with having LBP. As expected, LBP prevalence was associated with age, similar to other studies [1,9]. However, this study found no association between gender and LBP. Despite the fact that the female gender is known to be associated with having pain in many places in the body [9,10], in 2015, Alshami found gender to have a weak-to-no relationship with neck and back pain, respectively [11]. Other cultural and psychosocial factors can confound the relationship between gender and pain, which needs to be considered and examined in future research.

Chronic LBP and arthritis are the top two health conditions in clinical settings [12,13]. Around 33% of people with arthritis reported LBP [14], and 34% of people with chronic LBP have arthritis [15]. The bidirectional relationship can explain the increase in these conditions in clinical settings. However, there is limited, clear evidence of LBP and arthritis interaction. Non-mechanical causes, such as rheumatologic origin, lead to increasing pain severity [16]; moreover, mechanical LBP was associated with low physical activities, psychological symptoms, and functional limitations [17], which are significant moderate associations in people with rheumatoid arthritis [18,19,20]. Although there are conflicting results of inflammatory indicators in people with LBP, most previous studies indicated inflammatory components in people with LBP [21]. Inflammatory biomarkers, such as cytokines, an indicator of arthritis [22], increased in people with LBP [21,22,23]. Our study was consistent with a large cohort study that showed prevalent LBP and arthritis in the same age group (i.e., 20 to 49 years), with no gender differences in individuals with arthritis [13]. Another study showed that moderate-to-severe LBP was associated with the Western Ontario and McMaster Universities Osteoarthritis Index (WOMAC), for knee location only [24]. A cohort study showed LBP predicts increased pain and limitations for people with osteoarthritis of the hip only [25]. It should be noted that all participants were asked about the presence of arthritis, whether or not they had LBP. Moreover, the study was not concerned with the etiology of the LBP. However, we were limited to identifying the arthritis types and locations, in which future studies are needed to investigate the complex relationship between LBP and arthritis in the Saudi sample.

Although the interactions between high blood pressure and pain sensitivity are still under investigation, potential mechanisms can explain these associations [26]. The activation of the hypothalamic-pituitary-adrenal axis and spinal reflexes because of pain stimuli, increases cortisol levels, peripheral resistance, heart rate, and stroke volume [26]. The long-term effects of these activations can lead to arterial hypertension due to gradual exhaustion of the pain inhibitory systems or gradual changes in the baroreceptor function [26]. Consistent with the main finding, high systolic blood pressure (>140 mmHg) was associated with musculoskeletal complaints for both genders [27]; however, this association was not significant when age, education, BMI, physical inactivity, diabetes mellitus, alcohol consumption, smoking, and a history of cardiovascular issues were controlled [28]. A recent study on the Saudi sample showed a positive association between hypertension and chronic pain after controlling age, gender, education level, serum cholesterol, and smoking status [29]. In a longitudinal study, people with knee osteoarthritis had more chance of developing hypertension than people without osteoarthritis [30]. The use of anti-hypertensive medications and the duration of hypertension were not controlled in our study, which can have potential effects on these associations. Contrary to previous studies that showed hypertension as inversely related to musculoskeletal pain, hypertension-associated hypalgesia can weaken this association via the pain sensitivity modulation [31,32].

Anemia is caused by a variety of factors, including blood loss, malnutrition, reduced red blood cell morphology, viral processes, iron deficiency, and chronic inflammation [33]. The pathophysiological rationale for anemia-related weakness, healing deficit, unpleasant feelings, and pain is explained by the insufficiency of hemoglobin to provide adequate oxygen to tissues and return carbon dioxide to the lungs. [34] Thus, anemia can be a predisposing factor for pain episodes in people with LBP. Our findings revealed anemia as the highest risk factor of LBP compared to other risk factors that have been investigated in this research project. Increasing the risk of osteoporosis, osteopenia, and osteomalacia in patients with sickle cell anemia can explain the bone mineral density abnormalities in this population [35]. However, we were limited to explaining this association’s underlying mechanisms in which investigating the biomarkers of anemia in people with LBP was imperative. On 122 patients with intervertebral disc degeneration, there were positive correlations between iron deficiency (accompanied by anemia) and the grades of disc degeneration, in which iron supplements showed a potential preventive strategy for this population [34]. The literature needs more molecular studies to explain the hemoglobin value relationships with various conditions of LBP.

The direct cause of fracture due to osteoporosis has already been widely documented [36]. Although there is evidence that osteoporotic LBP is associated with fractures, the diagnosis is not always obvious. Using magnetic resonance imaging (MRI) on 1042 patients, 62% of those with LBP had an old spinal fracture [37]. Acute LBP is linked with symptomatic vertebral fractures, but chronic LBP is often caused by skeletal deformities, joint incongruity, and muscle and tendon stress [38]. Other back pain disorders in persons with osteoporosis include spondylosis, disc disease, and bone metastases [38]. It has been suggested that osteoporotic fractures are often overlooked in persons living with chronic LBP [38]. Previous research has revealed an increased prevalence of osteoporosis and poor bone mineral density in persons who suffer from chronic LBP [39,40]. It should be mentioned that the patients’ emotional experiences with pain might provoke adopting avoidance behaviors [38]. Our results were based on self-reported osteoporosis and a history of fractures, both of which are more common among LBP patients. Therefore, there is a need to explore the psychosocial aspects of LBP related to osteoporosis and fracture history.

Diabetes, as a risk factor of LBP, is shown to have no significant association with the adults in the Saudi community. Likewise, the multiple linear regression indicates a similar result that diabetes does not predict the severity of LBP amongst the population group. This would mean that improving or worsening diabetes in the population does not necessarily lead to the corresponding improvement or worsening of their LBP condition. These results are consistent with the study of Heuch et al. [41], which found no association between LBP and diabetes in men; however, in their study, LBP was an investigated risk factor of diabetes rather than the other way around. Conversely, several studies reported significant associations between diabetes and LBP [41,42,43,44]. In another study by Heuch et al. [42], diabetes and LBP were correlated in men but not in women. In Pico-Espinosa et al. [43], individuals with diabetes were found to have a higher risk of LBP. Dario et al. [44] also found an association between LBP and the prevalence of diabetes. All of these studies disagree with the result of the present study that diabetes and LBP are not significantly associated.

Moreover, the binary logistic regression reveals that cardiovascular diseases are not significantly associated with LBP in the population group. Similarly, multiple linear regression results suggest that cardiovascular diseases are not significantly associated with the severity of LBP in Saudi community adults. These results indicate that cardiovascular diseases do not necessarily cause LBP in Saudi community adults. In addition, the results denote that the sample data do not provide sufficient evidence that will allow the study to accept the hypothesis that cardiovascular diseases cause severity in LBP in Saudi community adults.

On the other hand, the other studies have mixed findings for the association between cardiovascular diseases and LBP. For example, Heliovaara et al. [45] found that atherosclerosis, a cardiovascular disease, is not etiologically linked to LBP, which is consistent with the results of this study. Kauppila [46] also revealed a weak association between cardiovascular risk factors and LBP. These results are, however, incongruent with the results of other studies. Furthermore, several studies revealed the contrary findings that cardiovascular diseases are significantly associated with LBP [47,48]. In a meta-analysis by Fayaz et al. [47], chronic LBP was strongly associated with cardiovascular diseases. All of these studies contradict the present research results that LBP and cardiovascular diseases are not significantly associated.

Furthermore, the results from the binary logistic regression show that dyslipidemia is not significantly associated with LBP. These results are, however, in contrast with some studies. For example, Kauppila [46] and Zafar et al. [49] revealed that dyslipidemia can lead to LBP as a result of aortic atherosclerosis. Likewise, Heuch et al. [50] revealed that abnormal serum lipid levels (related to dyslipidemia) are associated with an increased risk of low back pain severity. Furthermore, a positive correlation was found between lumbar disc herniation and serum lipid levels [51]. Thus, these studies negate the results of the present studies that suggest that dyslipidemia is not associated with the presence of LBP in Saudi community adults.

This work significantly contributes to the limited literature existing on the prevalence of low back pain in Saudi Arabia. In addition, we were able to examine the associated risk factors with LBP. However, our study has several limitations. First, it is a cross-sectional study; therefore, we could not establish the causality relationship between risk factors and LBP. Second, the small and geographical limited sample size can limit the generalizability of our results to different areas in Saudi Arabia. Therefore, future studies can include a larger sample size from other geographical regions to estimate LBP prevalence in the general population. Finally, a longitudinal study design is needed to better understand the relationship between associated risk factors and LBP in Saudi adults.

## 5. Conclusions

Low back pain is highly prevalent in the Saudi adult community, with more than a fourth of the sample reporting back pain. In addition, the research identified numerous modifiable risk factors (BMI, education, employment status, marital status, and smoking status) and unmodifiable risk factors (arthritis, hypertension, anemia, osteoporosis, and history of fractures) associated with low back pain in the study sample. This study calls for action to develop preventive, education, and management programs in society.

## Figures and Tables

**Table 1:** Demographics and clinical characteristics for participants.

Factors	Back Pain (*n* = 77)	No Back Pain (*n* = 199)	*p*-Value *
Age, years (mean ± SD) **	50.78 ± 8.15	48.66 ± 7.9	**0.021**
Sex (females) (% of back pain group)	67 (33.7%)	21 (27.3%)	0.38
BMI, Kg/m (mean ± SD) **	29.05 ± 5.5	28.83 ± 47	0.70
Marital status			0.54
Single	2 (2.6%)	12 (6.0%)	
Married	179 (89.9%)	70 (90.9%)	
Divorced	3 (3.9%)	4 (2.0%)	
Widowed	2 (2.6%)	4 (2.0%)	
Education			0.76
None	3 (3.9%)	13 (6.5%)	
Elementary	7 (9.2%)	11 (5.5%)	
Middle	9 (11%)	26 (13%)	
Secondary	20 (26.0%)	53 (26.6%)	
University	38 (49.4%)	96 (48.2%)	
Employment status			0.57
Unemployed	8 (10.4%)	30 (15.1%)	
Employed	52 (67.5%)	130 (65.3%)	
Retired	17 (22.1%)	39 (19.6%)	
Smoking (yes)	15 (19.5%)	33 (16.6%)	0.59
Arthritis (yes)	16 (21.3%)	22 (11.1)	0.048
Diabetes (yes)	16 (20.8%)	33(16.6%)	0.48
Hypertension (yes)	17 (22.1%)	19 (9.5%)	0.009
Cardiovascular disease (yes)	3 (3.9%)	6 (3.0%)	0.70
Dyslipidemia (yes)	13 (16.9%)	18 (9.0%)	0.08
Anemia (yes)	10 (13.0%)	5 (2.5%)	0.001
Osteoporosis (yes)	9 (11.7%)	4 (2.0%)	0.002
History of fractures (yes)	19 (24.7%)	16 (8.0%)	0.001
Back pain severity (mean) **	4.35 ± 2.19	NA	NA

* *p*-value was based on the chi-square for categorical variables or the Mann–Whitney test for continuous variables **. BMI: Body Mass Index.

**Table 2:** Binary logistic regression for low back pain versus risk factors.

Factors	OR (95% CI)	*p*-Value
History of fractures	3.93 (1.83, 8.44)	<0.001
Anemia	7.27 (2.06, 25.69)	0.002
Osteoporosis	5.94 (1.63, 21.69)	0.007
Arthritis	2.47 (1.12, 5.32)	0.024
Hypertension	2.34 (1.08, 5.05)	0.030
Dyslipidemia	1.92 (0.84, 4.39)	0.12
Diabetes	1.17 (0.57, 2.40)	0.67
Cardiovascular diseases	1.12 (0.25, 4.99)	0.88

OR: Odds Ratio. The covariates included age, gender, BMI, education, employment status, marital status, and smoking status.

**Table 3:** Multiple linear regression for the severity of low back pain and the associated risk factors.

Factors	B (95% CI)	*p*-Value
Osteoporosis	2.50 (1.18, 3.81)	<0.001
History of fractures	1.67 (0.87, 2.48)	<0.001
Arthritis	1.31 (0.51, 2.12)	0.001
Anemia	2.11 (0.85, 3.36)	0.001
Hypertension	1.11 (0.28, 1.95)	0.009
Dyslipidemia	0.73 (-0.16, 1.62)	0.11
Diabetes	0.22 (−0.53, 0.97)	0.55
Cardiovascular diseases	−0.44 (−2.00, 1.12)	0.57

B: Unstandardized coefficients. The covariates included age, gender, BMI, education, employment status, marital status, and smoking status.

## Data Availability

The data presented in this study are available on request from the corresponding author.
